# Testicule non descendu chez l’enfant: aspects épidémiologique, diagnostique et thérapeutique dans trois hôpitaux de référence de Douala, Cameroun

**DOI:** 10.11604/pamj.2024.49.70.40517

**Published:** 2024-11-08

**Authors:** Frantz Guy Epoupa Ngalle, Axel Stéphane Nwaha Makon, Willy Elysée Kana, Landry Oriole Mbouché, Armel Quentin Essomba, Pauline Mantho, Dieudonné Feukam, Edouard Hervé Moby Mpah, Faustin Mouafo Tambo, Marcellin Ngowe Ngowe

**Affiliations:** 1 Unité d'Urologie, Département de Chirurgie et Disciplines Affinitaires, Hôpital Général de Douala, Douala, Cameroun,; 2 Département de Chirurgie et Spécialités, Faculté de Médecine et de Sciences Biomédicales, Université de Yaoundé I, Yaoundé, Cameroun,; 3 Service d'Urologie, Hôpital Laquintinie de Douala, Douala, Cameroun,; 4 Département de Chirurgie et Spécialités, Faculté de Médecine et de Sciences Pharmaceutiques de Douala, Université de Douala, Douala, Cameroun,; 5 Service de Chirurgie Pédiatrique, Hôpital Laquintinie de Douala, Douala, Cameroun,; 6 Service d'Urologie, Hôpital de la Région Militaire de Douala n°2, Douala, Cameroun

**Keywords:** Testicule non descendu, cryptorchidie, orchidopexie, Undescended testicle, cryptorchidism, orchidopexy

## Abstract

**Introduction:**

le testicule non descendu (TND) désigne l'absence réelle d'un ou deux testicules de sa position normale dans le scrotum. Il peut entraîner l'atrophie testiculaire, sa cancérisation et l'infertilité masculine. Notre étude met en évidence les aspects épidémiologique, diagnostique et thérapeutique du TND dans 3 hôpitaux de référence de Douala.

**Méthodes:**

nous avons mené une étude descriptive et rétrospective sur 10 ans (1^er^ janvier 2012 au 31 décembre 2021). Nous avons colligé les dossiers des patients de 1 à 15 ans, opérés pour TND. Les données sociodémographiques, cliniques, paracliniques, thérapeutiques ont été collectées puis enregistrées et analysées avec les logiciels CS Pro 7.3 et SPSS 23.

**Résultats:**

nous avons répertorié 741 dossiers, exclus 595 car non pris en charge durant notre période d'étude. 105 ont été inclus. Les TND représentaient 1,39% (741 cas sur 53431 cas) des consultations urologiques. L'âge moyen était 6,65±3,13 ans. La vacuité scrotale était le principal motif de consultation (81,9%), découverte par un parent à domicile dans 76,7% des cas (n=66). Six virgule sept pour cent (6,7%) des patients (n=7) avaient un frère ayant un antécédent de TND et 2,8% (n=3) un père. Le testicule gauche était le plus représenté: 44,8% (n=47). Le testicule était palpable en inguinal dans 91,4% des cas (n=96). Le diagnostic était le plus souvent clinique. L'échographie était réalisée chez 14 patients (13,4%). La cryptorchidie était la plus diagnostiquée: 85,7% (n=90). La durée moyenne d'hospitalisation était de 1,85±0,74 jours. Deux voies d'abord sur le plan chirurgical ont été utilisées: inguinale 99 patients (94,3%) et coelioscopique 6 patients (5,7%).

**Conclusion:**

au terme de notre travail nous pouvons dire que la TND est une pathologie pour la moins fréquente en consultation urologique d'où la nécessité de savoir bien la diagnostiquer et la prendre en charge. Dans cette optique nous avons deux voies d'abord sur plan chirurgical. Inguinale la plus utilisée et la chirurgie laparoscopique essentielle si les testicules sont intra abdominaux ou tout simplement non palpables dans la région inguinale.

## Abstract (en)

**Introduction:**

Undescended testis (UDT) refers to the actual absence of one or both testicles from their normal position in the scrotum. It can lead to testicular atrophy, malignancy and male infertility. Our study highlights the epidemiological, diagnostic and therapeutic aspects of UDT in 3 referral hospitals in Douala.

**Methods:**

we conducted a descriptive and retrospective study over 10 years (January 1, 2012 to December 31, 2021). We collected data from the medical records of patients aged 1 to 15 years, who underwent surgery for UDT. The sociodemographic, clinical, paraclinical and therapeutic data were collected, recorded and analyzed using CS Pro 7.3 and SPSS 23 software.

**Results:**

we reviewed 741 records, excluding 595 that were not managed during our study period, leaving 105 cases included. UDT accounted for 1.39% (741 out of 53,431 cases) of urological consultations. The average age was 6.65±3.13 years. Scrotal emptiness was the main reason for consultation (81.9%), discovered by a parent at home in 76.7% of cases (n=66). Six point seven percent of the patients (n=7) had a brother with a history of UDT and 2.8% (n=3) a father. The left testis was most commonly affected: 44.8% (n=47). The testis was palpable in the inguinal region in 91.4% of cases (n=96). The diagnosis was mainly clinical, with ultrasound performed in 14 patients (13.4%). Cryptorchidism was the most diagnosed condition: 85.7% (n=90). The average hospital stay was 1.85±0.74 days. Two surgical approaches were used: inguinal in 99 patients (94.3%) and laparoscopic in 6 patients (5.7%).

**Conclusion:**

from our study, we can conclude that UDT is a relatively uncommon condition in urological consultations, emphasizing the importance of proper diagnosis and management. Surgically, two approaches are available: the inguinal approach, which is the most commonly used, and laparoscopy, which is essential when the testicles are intra-abdominal or non-palpable in the inguinal region.

## Introduction

Le testicule non descendu (TND) est la malformation urogénitale la plus courante chez le nouveau-né masculin [1]. Il désigne en effet, l'absence réelle d'un testicule (ou des 2) de sa position normale dans le scrotum. C'est une anomalie congénitale fréquente. Elle est plus souvent diagnostiquée et traitée pendant l'enfance [2]. L'incidence est variable et dépend de l'âge, affectant 1 à 4,6% des nouveau-nés à terme et de 1,1 à 45% des nouveau-nés prématurés [1]. Les TND sont classés dans deux grands groupes: les TND palpables et les TND non palpables. Les testicules palpables comprennent les testicules inguinaux et certains testicules ectopiques. Les testicules non palpables comprennent les testicules intra-abdominaux, absents, et parfois aussi quelques testicules ectopiques. Bien qu'absent dans les bourses, notons qu'environ 80% sont palpables (le plus souvent dans la région inguinale) [3]. Les conséquences engendrées par un TND sont multiples mais deux figurent au premier plan: risque d'infertilité et de cancérisation du testicule [4,5]. Le diagnostic repose sur l'anamnèse et l'examen clinique [6]. Le diagnostic définitif précisant le type de TND est parfois posé après l'intervention chirurgicale [7]. Dans notre contexte d'exercice africain, l'âge du diagnostic et de l'intervention reste le plus souvent supérieur à 2 ans [8]. Le traitement doit idéalement débuter à l'âge de 6 mois ou au plus tard à 18 mois [9]. La problématique que pose cette affection est liée à l'insuffisance diagnostique en bas âge par les cliniciens, la consultation tardive en cas de vacuité scrotale avec prise en charge tardive. Le but de notre travail était de décrire les profils sociodémographique, clinique et thérapeutique des TND dans la ville de Douala. Plus précisément, il s'agissait de: déterminer les variables sociodémographiques du TND dans la ville de Douala, de décrire la présentation clinique et les examens paracliniques utilisés et les moyens thérapeutiques utilisés et de donner l'évolution post-opératoire à court terme.

## Méthodes

**Type et cadre d'étude:** c'est une étude rétrospective et descriptive sur 10 ans (1^er^ janvier 2012 au 31 décembre 2021). Nous avons collecté les données pendant 6 mois (1^er^ décembre 2021 au 31 mai 2022). Nos sujets, tous opérés pour TND dans les services de chirurgie pédiatrique et d'urologie de trois hôpitaux de première et deuxième catégorie de la ville de Douala: Hôpital Général de Douala (HGD), Hôpital Laquintinie de Douala (HLD) et Hôpital Militaire Région n°2 de Douala (HMR2).

**Sélection des échantillons:** nous avons colligé de façon non exhaustive les dossiers des patients âgés de 1 à 15 ans. Nous avons exclu de notre étude les patients ayant des dossiers inexploitables et ceux avec le diagnostic de TND quelle qu'en soit la cause, mais non pris en charge dans nos 03 hôpitaux de recherche et pendant la période d'étude.

**Conception de l'étude:** les variables collectées étaient sociodémographiques, cliniques, paracliniques, thérapeutiques et évolutives permettant de déterminer la part des TND dans les consultations mais également les circonstances sociales, de découvertes et modalités de traitement.

**Analyse des données:** les résultats ont été enregistrés et analysés à l'aide des logiciels CS Pro 7.3 et IBM SPSS 23. Le test de chi carré a été utilisé avec un seuil de significativité inférieur à 0,05.

**Clairance éthique:** le Comité Institutionnel de Recherche de l'Université de Douala nous a délivré la clairance n°3078 CEI-Udo/05/2022/T pour cette étude.

## Résultats

**Figure 1:**
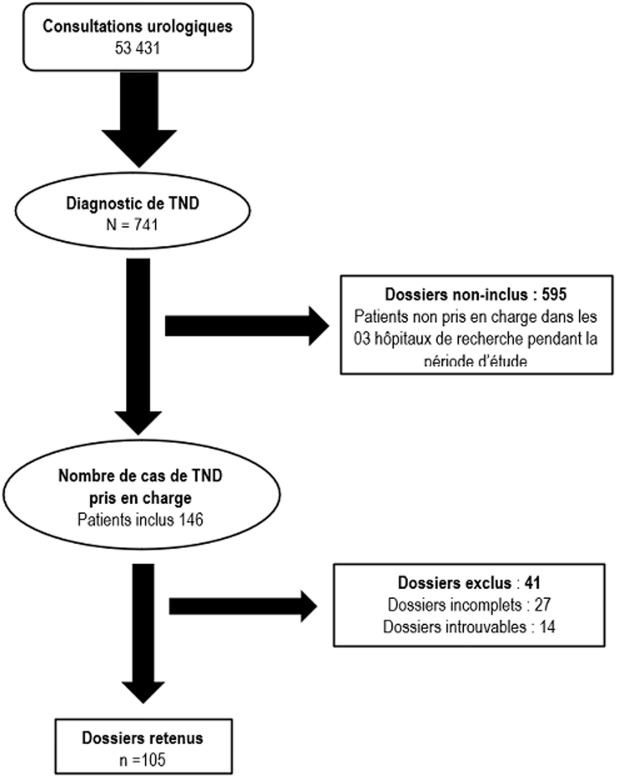
diagramme de flux de la population d'étude

**Figure 2:**
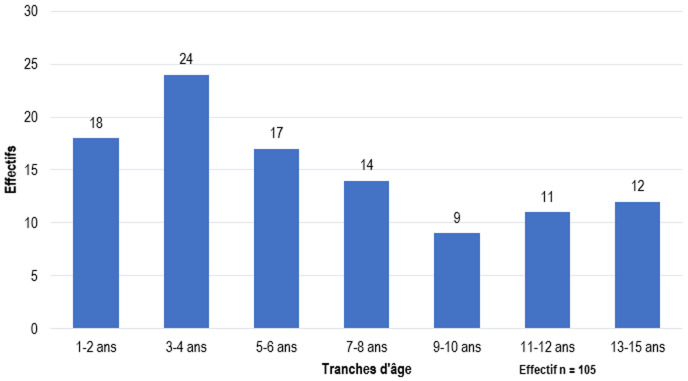
répartitions en fonction de l'âge

**Figure 3:**
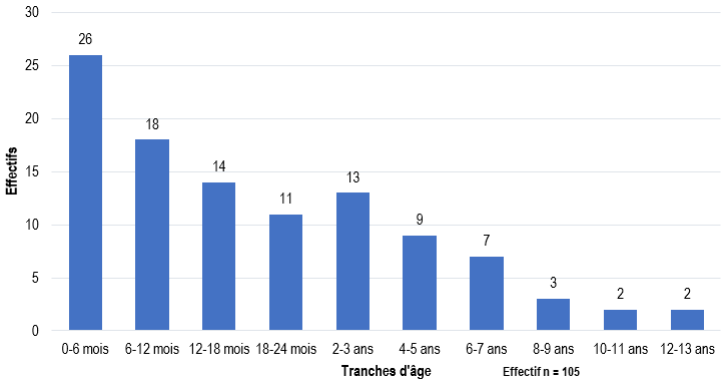
répartition en fonction de l'âge de constatation

**Aspects épidémiologiques:** pour la période d'étude, 53431 (tout sexe confondu) consultations urologiques ont été faites dans nos hôpitaux d'étude et nous avons ainsi répertoriés 741 dont le diagnostic était le TND (Figure 1). Soit une prévalence de 1,39%. Parmi eux, seulement 105 dossiers de malades ont été analysés et 636 dossiers rejetés pour insuffisance de données. L'âge moyen de prise en charge était de 6,7±3,1 ans avec des extrêmes allant de 1 à 15 ans (Figure 2). La tranche d'âge de 3 à 4 ans était la plus représentée avec (n=24). L'âge moyen de constatation de la vacuité scrotale était de 1,8±1,0 ans avec des extrêmes allant de la naissance à l'âge de 13 ans (Figure 3).

**Aspects diagnostiques:** les découvertes de cette affection se faisaient principalement entre la naissance et le 6^e^ mois (n=26). Le principal motif de consultation était la vacuité scrotale, répertoriée chez 86 patients (81,9%). La vacuité scrotale avait été constatée par un parent à domicile dans 76,7% de cas (n=66). Dans 17,5% de cas (n=15), elle avait été objectivée par un médecin lors des consultations post-natales. Seulement 3,5% de cas (n=3) avait été découvert au décours d'un examen systématique à la naissance. Un antécédent de prématurité a été retrouvé dans 3,8% de cas (n=4). Nous avons 6,7% des patients pris en charge (n=7) qui avaient un frère ayant un antécédent de TND. Le testicule gauche était le plus concerné avec une fréquence de 44,8% (n=47). Le testicule était majoritairement palpable à l'anneau inguinal superficiel dans 91,4% de cas (n=96). Dans la majorité des cas, le TND n'était associé à aucune anomalie des organes génitaux externes (OGE). Nous avons répertorié 8 cas d'hydrocèle (7,6%), l'hypospadias avait été retrouvé chez 3 patients (2,9%). L'examen clinique avait suffi à poser le diagnostic de TND dans 80,9% de cas (n=85). L'échographie avait été réalisée dans 13,4% de cas (n=14). La cœlioscopie diagnostique avait été utilisée chez 6 patients (5,7%). La cryptorchidie était le diagnostic le plus posé avec une fréquence de 86,7% de cas (n=91). Nous avons retrouvé un cas d'agénésie testiculaire (le diagnostic définitif était parfois posé après l'intervention chirurgicale par voie coelioscopique et inguinale). La cryptorchidie était le plus souvent prise en charge avant 7 ans, par rapport à l'ectopie testiculaire et le testicule oscillant qui eux étaient pris en charge plus tardivement, après l'âge de 7 ans (P=0,001). Le diagnostic de TND était isolé chez 57 patients (54,2%). Dans 23,9% de cas (n=25), le TND était associé à une hernie inguinale. Nous notons 2 cas (1,9%) de torsion du cordon spermatique sur testicule oscillant.

**Figure 4:**
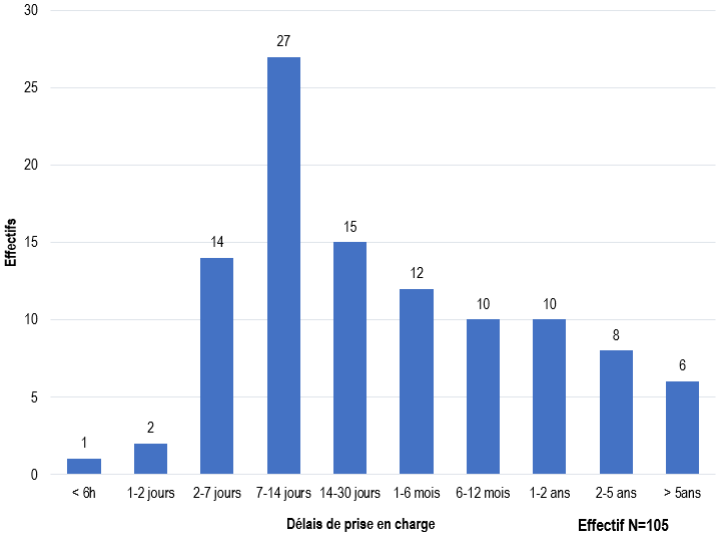
répartition en fonction du délai de prise en charge après le premier diagnostic médical

**Figure 5:**
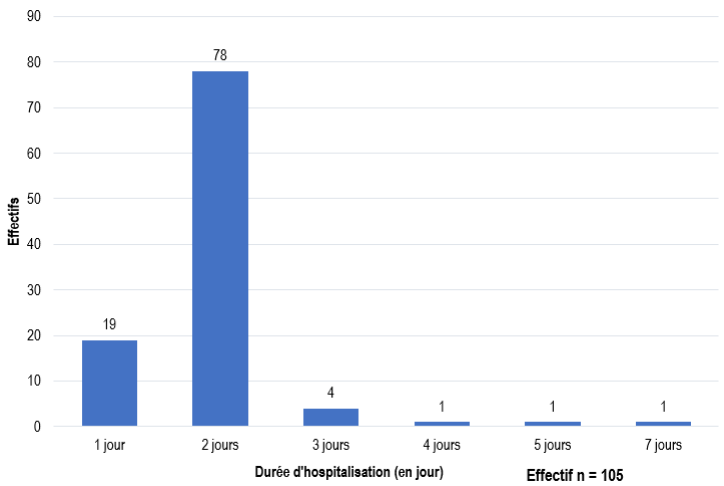
répartition en fonction de la durée d'hospitalisation

**Aspects thérapeutiques:** le délai moyen entre le premier diagnostic médical et la prise en charge était de 6,54±2,19 mois (Figure 4). Sur 3 patients arrivés en urgence (2 cas de torsion du cordon spermatique et 1 cas de traumatisme testiculaire sur TND), 1 seul avait été pris en charge dans les délais de 6h suivant le début de la symptomatologie. La voie d'abord inguino-scrotale était la plus réalisée avec une fréquence de 94,3% (n=99). La cœlioscopie avait été réalisée chez 6 patients (5,7%). Une hypotrophie testiculaire avait été retrouvée chez 17 patients (16,3%). On notait une persistance du canal péritonéo-vaginal chez 22 patients (21%) et une breveté du pédicule spermatique chez 32 patients (30,5%). Le geste chirurgical posé consistait à un abaissement suivi d'une orchidopexie. Une orchidectomie avait été pratiquée chez 2 patients (2%). Il s'agissait d'une nécrose testiculaire consécutive à une torsion du cordon spermatique et d’un cas de tumeur testiculaire sur TND. La durée moyenne d'hospitalisation était de 1,85±0,74 jours (Figure 5).

## Discussion

Dans cette d'étude, le TND représentait 1,39% des consultations urologiques. L'âge moyen de prise en charge était de 6,7±3,1 ans avec des extrêmes allant de 1 à 15 ans. Le motif principal de consultation était la vacuité scrotale retrouvée dans 86 cas (81,9%). Quatre-vingt-onze virgule quatre pour cent (91,4%) (n=96) de testicules étaient palpables et la majorité, soit 76% (n=73) étaient palpable à l'anneau inguinal superficiel. La cryptorchidie était en majorité représentée avec une fréquence de 85,7% (n=90). Le diagnostic est essentiellement clinique et dans 13,4% (n=14) des cas l'échographie concordait avec la clinique. Cinq virgule sept pour cent (5,7%) (n=6) des patients avaient bénéficié de la cœlioscopie. Le délai moyen d'hospitalisation était de 1,85±0,74 jours. L'évolution post-opératoire était bonne dans 94,3% de cas (n=99).

La prévalence du TND est assez variable selon les études et selon l'âge. Dans notre population d'étude, le TND représentait 1,39% des consultations urologiques. Nos résultats se rapprochent de la prévalence retrouvée par Gueye *et al*. au Sénégal qui avaient retrouvé une fréquence de 1,41% [10]. L'âge moyen de prise en charge était de 6,7±3,1 ans avec des extrêmes allant de 1 à 15 ans; ce qui est plus tardif par rapport à l'âge retrouvé par Oulad *et al*. au Maroc en 2018 (2,6 ans) [11]. Ce retard de prise en charge chirurgicale dans notre contexte a également été observé par Ndour *et al*. au Sénégal avec un âge moyen de 5,7 ans [8]; un retard qui pourrait s'expliquer par l'ignorance de l'usage des moyens chirurgicaux comme maillon fort du traitement, il pourrait également s'expliquer par un déficit de moyens financiers étant donné que la chirurgie a un coût non négligeable. Nous pouvons également noter que le retard de consultation du fait des croyances traditionnelles et/ou de la méconnaissance de la prise en charge par le personnel de santé.

La vacuité scrotale retrouvée dans 86 cas (81,9%) était le principal motif de consultation. Bouya *et al*. à Brazzaville avaient retrouvé 77,3% dans une étude similaire [12]. Cette vacuité scrotale est source d'angoisse parentale du fait de l'importance que revêt la fertilité dans notre milieu, ce qui pourrait expliquer la récurrence de ce motif de consultation. Seulement 3,5% de cas (n=3) avait été découvert au décours d'un examen systématique à la naissance. Cette faible découverte du TND lors de l'examen systématique à la naissance pourrait démontrer la difficulté éprouvée à examiner correctement les bourses d'un nouveau-né lors de l'examen clinique néonatal et ceci dû probablement à l'absence de reflexe d'examiner systématiquement les bourses à la naissance. Dans notre étude, 91,4% (n=96) de testicules étaient palpables et la majorité, soit 76% (n=73) étaient palpable à l'anneau inguinal superficiel. Grapin *et al*. en France avaient obtenu des résultats se rapprochant du nôtre. Les TND avaient été palpés dans 68,3% à l'anneau inguinal superficiel [13]. Selon la littérature, le type de TND le plus retrouvé est la cryptorchidie [14,15]. Nos résultats concordants à la littérature, nous ont permis d'établir que la cryptorchidie était en majorité représentée avec une fréquence de 85,7% (n=90). Des anomalies associées aux TND sont multiples; nous avons pu établir que la hernie inguinale était la plus représentée et répertoriée chez 25 patients (23,9%). D'autres anomalies urogénitales peuvent être présentes telles que l'hypospadias, qui est 10 fois plus fréquente que dans la population normale selon Gruner *et al*. [16]. Dans notre population d'étude, elle avait été retrouvée chez 3 patients (2,9%). Le diagnostic est essentiellement clinique (les explorations peuvent se discuter en cas de testicules non palpables au niveau inguinal). Dans notre étude, une échographie avait été réalisée chez 14 patients (13,4%) et qui majoritairement concordait avec la clinique. Ce résultat se rapproche de celui de Le Bartz *et al*. qui retrouvaient 8 cas sur 19 de testicules non palpables dont l'échographie n'avait pas été concordante avec l'anatomie de la malformation (environ 45% des cas). Les erreurs étant soit, des faux positifs (testicule visualisé, mais en fait absent), soit des faux négatifs (testicule non vu, mais en fait présent) [17].

Dans notre population d'étude, seulement 6 patients (5,7%) avaient bénéficié de la cœlioscopie. Boukli *et al*. retrouvaient jusqu'à 52,3% de patients ayant bénéficié de la cœlioscopie entre 2007 et 2016 au CHU d'Oran en Algérie [18]. Cette faible utilisation de la cœlioscopie dans notre contexte peut s'expliquer par un défaut de plateau technique adéquat, à son coût élevé et au faible nombre de praticiens formés en chirurgie laparoscopique. L'association d'une persistance du canal péritonéo-vaginal (PCPV) au TND est variable selon les études pouvant aller de 13 à 80% dans certaines études [19,20]. Ndour *et al*. retrouvaient 75% de PCPV dans une étude pareille au Sénégal en 2015 [8]. Dans notre série, la PCPV était associée au TND dans 21% de cas (n=22). Cette association fréquente peut s'expliquer par le fait que la non fermeture spontanée du CPV à la naissance chez certains enfants entraine la rétention mécanique du testicule dans le trajet péritonéo-vaginal. Le délai moyen d'hospitalisation était de 1,85±0,74 jours, aucun décès n'avait été enregistré et dans 94,3% de cas (n=99), l'évolution post-opératoire était bonne. Nos résultats sont concordants avec ceux de Bouya *et al*. qui chez 150 patients traités par orchidopexie à Brazzaville, retrouvaient 92,6 % de bons résultats [12].

Dans cette étude, nous n'avons pas pu explorer la coelioscopie comme intervention thérapeutique dû au faible taux de pratique dans les structures sanitaires concerné par l'étude. Nous avons pu explorer les aspects épidémiologiques, diagnostiques et thérapeutiques des patients grâce à une bonne documentation des dossiers médicaux.

## Conclusion

Au terme de notre travail, nous pouvons dire que la TND est une pathologie pour la moins fréquente en consultation urologique avec 1,39% de nos consultations urologiques, d'où la nécessité de savoir bien la diagnostiquer et la prendre en charge. Dans cette optique nous avons deux voies d'abord sur le plan chirurgical. Inguinale est la voie plus utilisée et la chirurgie laparoscopique essentielle si les testicules sont intra abdominaux ou tout simplement non palpables dans la région inguinale. Les médecins doivent être sensibilisés sur l'importance d'examiner les bourses des nouveau-nés durant leur consultation systématique à la naissance.

### Etat des connaissances sur le sujet

- Pas d'études réalisées sur le profil sociodémographique, clinique et thérapeutique dans la ville de Douala au Cameroun;
- Faible prévalence des TND en consultation urologique;
- Prise en charge chirurgicale précoce des TND.

### Contribution de notre étude à la connaissance

- Montrer le profil épidémiologique des TND dans la ville de Douala particulièrement et dans la région d'Afrique centrale dont le Cameroun fait partie car il n'y a pas beaucoup d'études dans la sous-région;
- Le diagnostic est le plus souvent clinique dans notre étude;
- Amélioration des stratégies de prise en charge des TND.

## Remerciements

Nous adressons nos remerciements à l'administration et le personnel des services de chirurgie générale de l'Hôpital Général de Douala, des services d'urologie et de chirurgie pédiatrique de l'Hôpital Laquintinie de Douala et le service d'urologie de l'Hôpital de la Région Militaire n°2.
